# DAVID: An open-source platform for real-time transformation of infra-segmental emotional cues in running speech

**DOI:** 10.3758/s13428-017-0873-y

**Published:** 2017-04-03

**Authors:** Laura Rachman, Marco Liuni, Pablo Arias, Andreas Lind, Petter Johansson, Lars Hall, Daniel Richardson, Katsumi Watanabe, Stéphanie Dubal, Jean-Julien Aucouturier

**Affiliations:** 1 Science & Technology of Music and Sound (STMS), UMR 9912 (CNRS/IRCAM/UPMC), 1 place Stravinsky, 75004 Paris, France; 2 Inserm U 1127, CNRS UMR 7225, Sorbonne Universités UPMC Univ Paris 06 UMR S 1127, Institut du Cerveau et de la Moelle épinière (ICM), Social and Affective Neuroscience (SAN) Laboratory, Paris, France; 3 Lund University Cognitive Science, Lund University, Lund, Sweden; 4 Swedish Collegium for Advanced Study, Uppsala, Sweden; 5 Department of Experimental Psychology, University College London, London, UK; 6 Department of Intermedia Art and Science, Faculty of Science and Engineering, Waseda University, Tokyo, Japan; 7 Research Center for Advanced Science and Technology, The University of Tokyo, Tokyo, Japan

**Keywords:** Emotional transformations, Nonverbal behavior, Voice, Real-time, Software, Infra-segmental cues

## Abstract

We present an open-source software platform that transforms emotional cues expressed by speech signals using audio effects like pitch shifting, inflection, vibrato, and filtering. The emotional transformations can be applied to any audio file, but can also run in real time, using live input from a microphone, with less than 20-ms latency. We anticipate that this tool will be useful for the study of emotions in psychology and neuroscience, because it enables a high level of control over the acoustical and emotional content of experimental stimuli in a variety of laboratory situations, including real-time social situations. We present here results of a series of validation experiments aiming to position the tool against several methodological requirements: that transformed emotions be recognized at above-chance levels, valid in several languages (French, English, Swedish, and Japanese) and with a naturalness comparable to natural speech.

## Introduction

The use of well-defined stimulus material is an important requirement in experimental research, allowing for replicability and comparison with other studies. For this reason, researchers interested in the perception of emotions often use datasets of stimuli previously validated with affective norms. An increasing number of such datasets exist for both facial expressions (e.g., the Karolinska Directed Emotional Faces - 70 individuals, each displaying seven facial expressions, photographed from five different angles, Goeleven et al., 2008), vocal expression (e.g., the Montreal Affective Voices - ten actors, each recording nine non-verbal affect bursts, Belin et al., 2008) and musical extracts (e.g., The Montreal Musical Bursts - 80 short musical improvisations conveying happiness, sadness, or fear, Paquette et al., 2013).

However, using datasets of static stimuli, regardless of how well controlled, comes with a number of generic limitations. First, such datasets leave researchers only little control over the para-emotional parameters of the expressions (e.g., which specific person is expressing the emotion or what verbal content accompanies the expression), while some research questions may require more control over the stimulus material: for instance, to investigate social biases, one may want a certain emotion to be expressed by members of two different social groups with the exact same acoustic cues (see e.g., Neuberg 1989). Second, actor-recorded stimuli do not allow for fine control over the intensity with which emotions are expressed: e.g., some actors may be more emotionally expressive than others, or perhaps more expressive when it comes to happiness than sadness (see e.g., Wallbott 1988). In an attempt to control for such parameters, various researchers have used morphing techniques between e.g., a neutral and an emotional facial expression (Sato et al. 2004), or between two different emotional vocal expressions (Bestelmeyer et al. 2012). Morphings can gradually increase the recognizability of an emotional stimulus or create arbitrarily ambiguous emotional voices (Bestelmeyer et al. 2012). However, they do not only affect expressive cues that are involved in the communication of emotion, but also cues that may not be linked directly to emotions, or that one may not want to be morphed. For instance, it requires the use of very advanced techniques to only morph the pitch, but not the loudness, between two emotional voices. Moreover, with morphings, the para-emotional context (e.g., specific speakers) remains limited to the stimuli that are included in the database. A final generic limitation of pre-recorded datasets is that they necessarily only consist of third-person stimuli. However, in many experimental contexts, one may desire to control the emotional expression of the participants themselves, and not that of unknown actors. For example, social psychology researchers may want to study participants’ interactive behavior while controlling whether they sound positive or negative. It remains difficult to create such situations without demand effect, e.g., not asking or otherwise leading participants to “act” happy or sad.

Rather than a data set of controlled emotional *stimuli*, it would therefore be useful to have a data set of controlled emotional *transformations*, that can be applied to arbitrary stimulus material while still preserving well-defined properties of recognizability, intensity and naturalness. Such data sets exist in the visual domain, for the synthesis of facial expressions. For instance, tools have been developed that can very precisely manipulate facial cues to alter perceived personality traits (Todorov et al. 2013) or the emotional expression (Roesch et al. 2011) of computer-generated or digitized faces, allowing for a high level of control. However, no such tools exist in the domain of vocal expression to the best of our knowledge. More precisely, while emotional voice synthesis is an active research field in the audio engineering community, no such tool comes with the experimental validation and technical requirements necessary for psychological research.

The human voice is a powerful medium for the expression of emotion (Bachorowski and Owren 1995; Juslin et al. 2005). With a suitable voice transformation tool, it should be possible to change the emotional expression of speech after it is produced and, if computed fast enough, the transformations could even appear to occur in “real time”. With such a tool, one would be able to modify vocal emotional expressions in live and more realistic settings and study not only the perception of emotions in third-party stimuli, but also the perception of self-produced emotions, opening up a vast amount of experimental questions and possibilities.

In this article, we present DAVID^1^, a novel open-source software platform providing a set of programmable emotional transformations that can be applied to vocal signals. The software makes use of standard digital audio effects, such as pitch shift and spectral filtering, carefully implemented to allow both realistic and unprecedentedly fast emotional transformations at the infra-segmental level of speech (“Emotional transformations”). DAVID was used in a previous study by Aucouturier et al. (2016) in which participants read a short text while hearing their voice modified in real time to sound more happy, sad, or afraid. Results of this study showed that a great majority of the participants did not detect the manipulation, proving that the emotional transformations sounded natural enough to be accepted as self-produced speech and that they were fast enough to allow for uninterrupted speech production. In addition, participants’ mood ratings changed in the same direction as the manipulation, suggesting that the transformations carry some emotional meaning.

Extending beyond this first experiment, we present here results from an additional series of experimental studies that aim to position the tool against four important methodological requirements for psychological and neuroscience research, namely that the transformations are recognizable, natural, controllable in intensity, and reasonably inter-cultural (see “Validation studies”). Based on these results, we then propose a list of application ideas in a selection of research areas where we argue this new transformation software will be of particular importance.

## Emotional transformations

### Emotional speech synthesis techniques

Consciously or not, we convey emotional information with our speech. The words and syntactic structures that we use reveal our attitudes, both towards the topic of conversation and towards the person we converse with. Besides words, the sole *sound* of our voice is rich in information about our emotional states: higher fundamental frequency/pitch when happy than sad (Scherer and Oshinsky 1977), faster speech rate when excited, raising intonation/prosody when surprised (? BAN06). Computerized audio analysis and synthesis are important techniques to investigate such acoustic correlates of emotional speech (Scherer 2003a). Widely used phonetical analysis tools like Praat (Boersma and Weenink 1996) allow the automatic analysis of large corpus of speech in terms of pitch, duration and spectral parameters (Laukka et al. 2005). More recently, speech synthesis techniques, typically pitch-synchronous overlap-and-add methods (PSOLA) and shape-invariant phase vocoder (Roebel 2010), support the active testing of hypotheses by directly manipulating the acoustic parameters of the vocal stimuli (Bulut and Narayanan 2008).

Beyond its use for psychological experimentation, emotional speech synthesis is now a widely researched technique per se, with applications ranging from more expressive text-to-speech (TTS) services for e.g., augmentative and alternative communication devices (Mills et al. 2014), restoration of voices in old movies (Prablanc et al. 2016) or more realistic non-player characters in video games (Marsella et al. 2013). One major concern with such systems is the degree of realism of the synthesized voice. In early attempts, this constraint was simply relaxed by designing applications that did not need to sound *like anyone in particular*: for instance, cartoon baby voices for entertainment robots (Oudeyer 2003). For more realism, recent approaches have increasingly relied on modifying pre-recorded units of speech, rather than synthesizing them from scratch (but see Astrinaki et al., 2012). One of such techniques, concatenative synthesis, automatically recombines large numbers of speech samples so that the resulting sequence matches a target sentence and the resulting sounds match the intended emotion. The emotional content of the concatenated sequence may come from the original speaking style of the pre-recorded samples (“select from the sad corpus”) (Eide et al. 2004), result from the algorithmic transformation of neutral samples (Bulut et al. 2005), or from hybrid approaches that morph between different emotional samples (Boula de Mareüil et al. 2002). Another transformation approach to emotional speech synthesis is the recent trends of “voice conversion” research, which tries to impersonate a target voice by modifying a source voice. This is typically cast as a statistical learning task, where the mapping is learned over a corpus of examples, using e.g., Gaussian mixture models over a parameter space of spectral transformation (Inanoglu and Young 2007; Godoy et al. 2009; Toda et al. 2012).

The tool we propose here, a voice transformation technique to color a spoken voice in an emotional direction which was not intended by the speaker, is in the direct tradition of these approaches, and shares with them the type of audio transformation used (i.e., temporal, pitch, and spectral) and the need for high-level quality. However, we attempt to satisfy a very different constraint: the transformed voice has to be a realistic example of its speaker’s natural voice. Previous approaches have attempted—and succeeded—to produce either a realistic third-person voice (e.g., a considerate newscaster - Eide et al., 2004) or an exaggerated first-person (e.g., me as a happy child, me as an angry monster - Mayor et al., 2009). We describe here a technique which synthesizes a *realistic first-person*: me when I’m happy, me when I’m sad. We refer to the transformation as “natural”, in that it effectively imparts the impression of a specific emotion for the listeners while being judged to be as plausible as other, non-modified recordings of the same speaker.

A second particularity of this work is that the transformation can be done in real time, modifying speech as it is uttered, without imparting any delay capable of breaking a natural conversation flow (in practice, less than 20ms). This differentiates from previous work in several ways. First, the expectation of high realism has compelled previous approaches to design increasingly sophisticated analysis methods - time-domain PSOLA, linear prediction PSOLA (Moulines and Charpentier 1990), linear-prediction time-scaling (Cabral and Oliveira 2005), wide-band harmonic sinusoidal modeling (Mayor et al. 2009), to name but a few. As a consequence, none of these approaches can meet real-time constraints, especially as predictive models require a short-term accumulator of past data (but see Toda et al., 2012; Astrinaki et al., 2012, for recent progress on that issue). Second, many techniques rely on strategies that are incompatible with the real-time following of an input voice: speeding the voice up or down, anticipating the end of a sentence to raise its prosody, or inserting paralinguistic events such as hesitation markers <ERR> or <AHEM>. The approach described here manages to operate in real-time by careful design rather than by technical prowess. First, we favor effects that can be implemented efficiently, such as simple time-domain filtering, and in cascade (such as vibrato and pitch shifting both using the same pitch shifting module). Second, because the manipulation is designed to be ‘‘natural”, our effects operate over very subtle parameter ranges (e.g., + / − 40 cents pitch shifting, instead of e.g., + / − 1 octave as targeted in Cabral and Oliveira 2005), for which even simplistic (and fast) approaches are sufficient.

An important consequence of this positioning of the tool is that its transformations only operate at the infra-segmental level of speech, i.e., on speech cues that can be manipulated on a phonemic basis, without taking account of the supra-segmental structure. These concern e.g., static pitch, amplitude, voice quality, and spectral content, but excludes other important cues for emotional expression such as prosody, speed or timing. For instance, varying speech speed is a commonly observed correlate of emotional voices (e.g., sad voices tend to be slower and happy voices faster - Scherer and Oshinsky, 1977), however playing speech faster in real time is impossible by construction and playing it slower would result in noticeable delays. Similarly, happy voice prosody tends to raise in pitch at the end of sentences (Bänziger and Scherer 2005; Hammerschmidt and Jurgens 2007), however manipulating this in real time requires to process larger segments of audio and anticipate structural boundaries, also with a consequent augmentation of the system’s latency (if feasible at all).

Because of the importance of infra-segmental cues in both the perception and production of vocal emotions (see e.g., Bachorowski and Owen 1995), we believe that the current tool is a simplified, but not meaningless, approximation of emotional speech. However, it is important to keep in mind that emotional expressions produced with the tool do not explore the full expressive space of authentic human-produced speech, or that of some of the alternative non-real-time speech synthesis systems.

### Software distribution

DAVID is a software platform developed to apply audio effects to the voice both online and offline. The platform provides four types of audio effects, or building blocks, that can be combined in different configurations to create several emotions: happy, sad, and afraid (and more are possible). DAVID is implemented as an open-source patch in the Max environment (Cycling74), a programming software developed for music and multimedia. The DAVID software and accompanying documentation can be downloaded under the MIT license from http://cream.ircam.fr. Using DAVID first requires to install the Max environment, which is provided in free versions for Windows and Mac systems. DAVID comes with the parameter presets used in the validation studies described below, but users also have full control over the values of each audio effect to create their own transformations and store them as presets for further use. The present article is based on software version v1.0 of DAVID (release date: 15/10/2015), see the DAVID website for further updates and new functionalities.

### Algorithms used in DAVID

DAVID is designed as a collection of building blocks, or “audio effects”, that can be combined in different configurations to create emotion transformations. Each audio effect corresponds to a frequently identified correlate of emotional voices in the literature (see reviews by Scherer 2003b; Juslin and Laukka 2003; Patel and Scherer 2013). For instance, fear is often associated with fluctuations in the voice pitch (Laukka et al. 2005; Dromey et al. 2015) - an effect we implement here as vibrato (see below). However, we choose not to associate an individual effect with an individual emotion (e.g., vibrato $\leftrightarrow $ fear), because we observed a large degree of overlap and/or contradicting claims in previous works. For instance, Laukka et al. (2005) observe that a low mean pitch is a correlate of positive valence, but also of negative arousal, casting doubt on what should be associated with a state of joy. Rather, audio effects in DAVID are best described as “things that often happen to one’s voice when in an emotional situation”. How these effects map to emotions depends on the way the effects are quantified, the way emotions are represented (words, multidimensional scales, etc.), and possibly other factors such as context or culture (Elfenbein and Ambady 2002), and elucidating this mapping is not the primary concern of our work.

In the experiments presented here, we tested three types of transformations - happy, sad and afraid - each composed of several, sometimes overlapping audio effects (e.g., afraid and happy both include the inflection effect). The audio effects used in each manipulation are listed in Table 1, and their algorithmic details given below.

**Table 1:** List of the atomic digital audio effects used in this work, and how they are combined to form emotional transformations happy, sad, and afraid

Effects		Transformations		
		Happy	Sad	Afraid
Time-varying	Vibrato			✓
	Inflection	✓		✓
Pitch shift	Up	✓		
	Down		✓	
Filter	High-shelf (“brighter”)	✓		
	Low-shelf (“darker”)		✓	

**Fig. 1:**
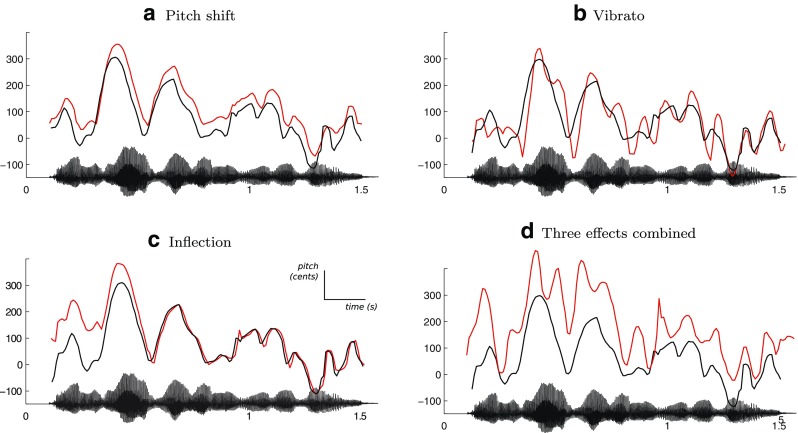
Three of the audio effects available in DAVID, applied on the same recording by a French female speaker, saying *“Je suis en route pour la réunion”* (I’m on my way to the meeting). The *solid black line* represents the time series of pitch values in the original recording (estimated with the SWIPE algorithm - Camacho and Harris 2008) and the *red line* represents the pitch of manipulated audio output. The speech waveform of the unmodified recording is shown on the *x*-axis of each subfigure. Pitch values on *y*-axis are normalized to cents with respect to mean frequency 200 Hz. **a** The pitch is shifted upwards by 40 cents. **b** Vibrato is applied with a rate of 8.5 Hz and a depth of 40 cents. **c** Inflection kicks in at the start of the utterance, with an initial shift of + 140 cents, and recedes after 500 ms (implemented in the happy transformation). **d** The three effects combined, for illustration purposes. The audio effects can be applied in any configuration

#### Pitch shift

Pitch-shift denotes the multiplication of the pitch of the original voice signal by a constant factor *α*. Increased pitch (*α* > 1) often correlates with highly aroused states such as happiness, while decreased pitch (*α* < 1) correlates with low valence, such as sadness (Scherer 2003b; Juslin and Laukka 2003; Patel and Scherer 2013).

##### Implementation

In DAVID, pitch-shift is implemented as a standard application of the harmonizer, i.e., a time-varying delay. For this, a maximum delay time has to be specified, that consequently defines the needed amount of memory in order to delay the incoming signal, and thus the latency of the algorithm (this parameter is accessible as *window* in DAVID). Pitch is shifted by a constant factor (see Fig. 1a). In order to reduce computational load, early processing stages of the constant pitch-shift algorithm are shared with the time-varying vibrato and inflection, and factors for multiplying pitch are accumulated where appropriate.

##### Parameters

Pitch-shift is used in the happy transformation with a positive shift of + 50 cents^2^ (i.e., one half of a semitone), and in the sad transformation with a negative shift of −70 cents. The maximum delay time is set by default to 10 ms.

#### Vibrato

Vibrato is a periodic modulation of the pitch (fundamental frequency) of the voice, occurring with a given rate and depth. Vibrato, also related to jitter, is frequently reported as a correlate of high arousal (Laukka et al. 2005) and is an important marker of emotion even in single vowels (Bachorowski and Owren 1995).

##### Implementation details

Vibrato is implemented as a sinusoidal modulation of the pitch shift effect, with a rate parameter (modulation frequency, in Hz), a depth (in cents) and a random variation of the rate (in percentage of the rate frequency). Figure 1b shows a typical output of the algorithm (using a speech extract from our experimental data).

##### Parameters

The afraid transformation uses vibrato with a rate of 8.5 Hz, a depth of + / − 40 cents and a 30% random rate variation.

#### Inflection

Inflection is a rapid modification (∼500 ms) of the pitch at the start of each utterance, which overshoots its target by several semitones but quickly decays to the normal value. The use of inflection leads to increased variation in pitch, which is associated with high emotional intensity and positive valence (Laukka et al. 2005). For instance, Pell and Kotz (2011) reported that expressions of happiness contain higher levels of pitch variation than expressions of fear, which in return comprise more pitch variation than expressions of sadness.

##### Implementation details

DAVID analyzes the incoming audio to extract its root-mean-square (RMS), using a sliding window. When the RMS reaches a minimum threshold, the system registers an attack, and starts modulating the pitch of each successive frame with a given inflection profile (see Fig. 1c). The inflection profile can be specified by the user, together with a minimum and maximum pitch shift, as well as a duration.

##### Parameters

Two inflection profiles are proposed: in the first, associated in our experiments to the happy transformation, pitch quickly increases from −200 cents to + 140 cents, then decaying to the original pitch over a total duration of 500 ms; the second, associated to the afraid effect, is a sinusoidal curve between −200 and + 200 cents with a duration of 500 ms, starting at its maximum position and decaying to the original pitch.

#### Filtering

Filtering denotes the process of emphasizing or attenuating the energy contributions of certain areas of the frequency spectrum. The acoustics of emotional speech are rarely analyzed in terms of global spectral changes (Tartter 1980; Pittam et al. 1990), however, we found that some simple filtering is often successful in simulating behaviors of vocal production that are typically associated with emotional content. For instance, high arousal emotions tend to be associated with increased high frequency energy, making the voice sound sharper and brighter (Pittam et al. 1990); this can be simulated with a high-shelf filter. Conversely, “sad” speech is often described as darker, a perception simulated with a low-shelf filter.

In addition, a recent study by Ma and Thompson (2015) showed that manipulations of the frequency spectrum of environmental sounds (human actions, animal sounds, machine noise and sounds in nature) changed their valence and arousal ratings; sounds with increased high-frequency content were perceived as more positive and more arousing than control both sounds and sounds with increased low-frequency content. Please note that the spectral modulations used by Ma and Thompson (2015) are not the same as the filters used in DAVID and that their stimuli did not comprise human speech per se. However, this study does illustrate how spectral characteristics of sounds can affect their emotional perception.

##### Implementation details

All filters are implemented as 5-order Butterworth IIR filters. Filter design is done offline (not in real-time), with a bilinear transform.

##### Parameters

The happy transformation uses a high-shelf filter with a cut-off frequency at 8000 Hz, + 9.5 dB per octave (“brighter”). The sad transformation uses a low-shelf filter with a cut-off frequency at 8000 Hz, −12 dB per octave (“darker”).

#### System and algorithm latency

**Fig. 2:**
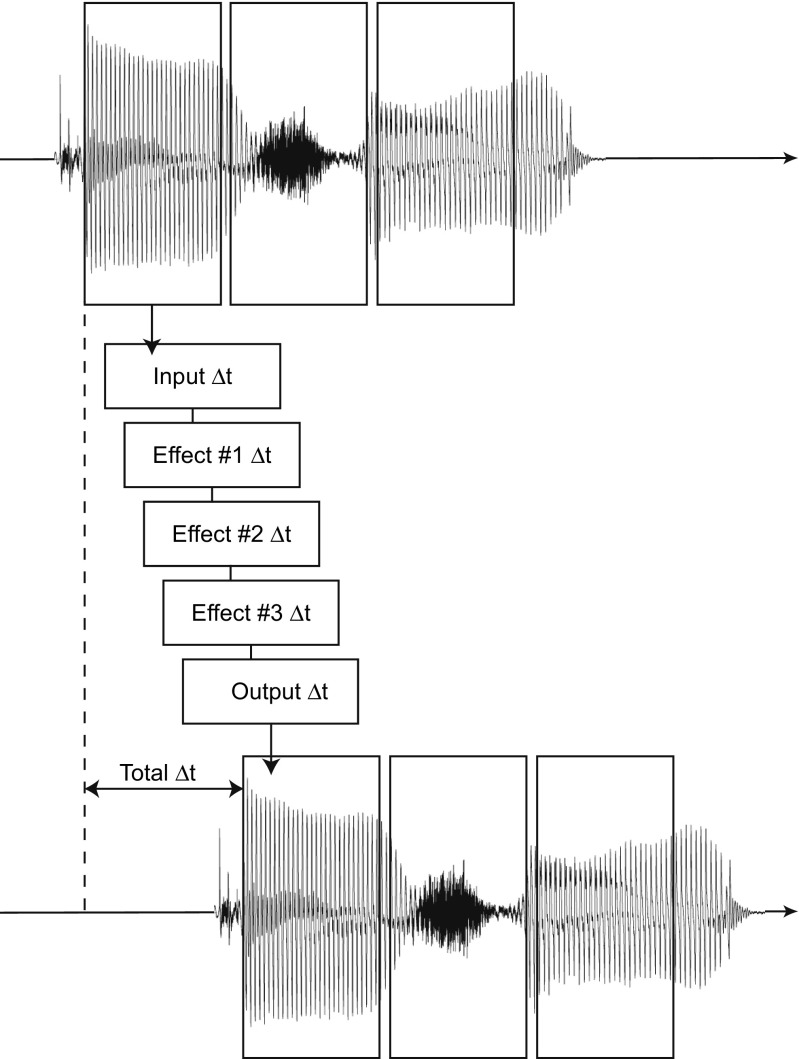
Illustration of the delays involved in the realization of our real-time audio processing system. Beyond a baseline I/O latency (input and output Δ_*t*_), each atomic effect in the signal data flow (3 as illustrated here) imparts further delay, which depends on the effect’s algorithmic complexity

Figure 2 gives a schematic explanation of the two types of latencies (round-trip and algorithmic) involved in the realization of our real-time audio processing system. The incoming audio has to be captured and converted from analog to digital format before reaching the memory of the application. This causes a first delay (input Δ_*t*_). Similarly, after all processing is done, the digital signal has to be routed back from application memory to the output device, undergoing digital to analog conversion - hence an output Δ_*t*_. Both input and output delays (the sum of which is known as *roundtrip* latency) occur even if no processing is done: this is the delay time that is typically experienced when talking into a microphone plugged into the sound card, while listening back to the voice through headphones. Roundtrip latency depends on the system’s hardware and software, and can be easily optimized to the range 2–5 ms (Wang et al. 2010). However, when some processing is applied, low latencies can degrade sound quality, because the high rate at which computer and audio card exchange data provides less samples than needed for some algorithms to achieve a correct result. In the Max environment, the exchange rate between the CPU and the sound card is controlled by means of the *I/O vector size* (which corresponds to the input and output Δ_*t*_), while the *signal vector size* determines the exchange rate within Max itself. Our recommended software set-up for using DAVID in a real-time context consists of a I/O vector size: 256 samples and a signal vector size: 128 samples.

The algorithmic latency is the delay added to the system’s round-trip latency and needed to run the audio transformations. All of the transformation modules in DAVID are based on the same pitch shifting engine, the harmonizer described in “Pitch shift”. The only latency is thus given by the maximum delay time in the harmonizer, which is set by default to 10 ms. This latency does not depend on the system’s hardware and software.

Our recommended hardware set-up therefore is a system which allows to run DAVID with a minimal round-trip latency, using the above vector sizes:

**Computer :** DAVID is implemented as an open-source patch for the (free, close-source) audio processing platform Max (Cycling’74). According to its seller, system requirements for Max7 are Intel Mac with Mac OS X 10.7 (or later), OR a PC with Windows 7 (or later); Multicore processor; 2 GB RAM; 1024 ×768 display. If a system widely departs from these specifications, one should consider installing earlier versions of the Max platform.
**Audio interface :** a medium to high-end external audio interface. Slower audio interfaces will degrade the round-trip latency, and thus the global latency. In this study, we used a RME UCX Fireface sound card, with which we consistently measured a roundtrip latency of 9.5 ms, and thus a global latency of 19.5 ms.

Note that the maximum acceptable delay depends on the context of the study. Aucouturier et al. (2016) found that vocal feedback with a latency of 20 ms did not disrupt continuous speech. However, in other settings, such as video calls, a longer delay may be acceptable.

In complement, audio equipment needed to run the system will depend on one’s application:

**Headphones :** If the application involves speakers hearing their transformed voice *while* they speak, it is desirable to acoustically isolate the participants from their own non-modified speech; otherwise, they may hear both their normal voice and, after a short delay, their manipulated voice. For this purpose, we recommend a closed-type, rather than open-type set of headphones. Additionally, because the manipulations affect voice spectrum, headphones should present a relatively flat frequency response. In this study, we used Beyerdynamic’s DT770 Pro headphones, which we found satisfy these requirements.
**Microphone :** If the application involves transforming a participant’s direct input through a microphone (rather than transforming pre-recorded audio files), using either a directional microphone or close-miking with an omnidirectional microphone is recommended to avoid that too much environmental noise and room reverberation is picked up and transformed along with the speaker’s voice. In this study, we used DPA d:fine 4066-F Headset microphones, which we found satisfied this constraint.

## Validation studies

We present here results from a series of experimental studies that aim to position the tool against four important requirements that we consider indispensable for it to be useful in psychological and neuroscience research:

1. The emotional tone of the transformed voice should be recognizable.
2. The transformed voices should sound natural and should not be perceived as synthetic.
3. The software user should be able to control the emotional intensity of the transformed voices.
4. The three criteria mentioned above should apply to several languages, making the tool applicable in multiple research environments, as well as to cross-cultural research questions.

### Stimuli

We recorded six neutral sentences spoken by 12 French (six female), nine English (four female), 14 Swedish (seven female), and 11 Japanese (seven female) speakers between 18 and 30 years of age. The sentences were chosen from a set of semantically neutral sentences (Russ et al. 2008). Speakers were asked to produce each sentence eight times with a neutral expression and three times with each of the emotional expressions (happy, sad, afraid). The recordings took place in a sound-attenuated booth, using GarageBand software (Apple Inc.) running on an Apple Macintosh computer and a headset microphone (DPA d:fine 4066) connected to an external sound card (RME UCX Fireface). Audio was acquired at a 44.1-kHz sampling rate and 24-bit resolution. Based on the quality of the recordings, six speakers (three female) and four sentences were selected in each language. Recordings were rejected in case of pronunciation errors and if there were clear recording artifacts, such as tongue clicks, breathing noise, microphone impact or pops. This selection was done based on the raw files, before manipulation with DAVID. The selected recordings were finally included in three behavioral experiments to test the validity of the software tool, yielding 24 different speaker-sentence combinations per language.

For each speaker and sentence, we selected the first four neutral recordings for further processing. If the quality was insufficient (rejection criteria were the same as stated above), we selected the next available recording. For the sentences spoken with an emotional tone, we selected only one recording.

Three out of the four neutral recordings were processed with our tool to transform the voices into happy, sad, and afraid voices. For each emotion, we selected the parameters for the audio effects such that we judged the emotional transformation to be recognizable, yet natural. In the remainder of this article, we will refer to these parameter settings, indicated in “Emotional transformations” and in Table 2, as the “nominal level”. Furthermore, we processed the recordings with the same audio effects at two increasingly reduced intensity levels. We thus tested three emotional transformations at three intensity levels. All audio files were normalized for maximum peak intensity using Audacity version 2.1.0. All stimuli used in this study are made available for download from https://archive.org/details/DAVIDAudioFiles

**Table 2:** List of the parameters used in the validation experiments. For the afraid transformation different values were used for male and female voices, due to strong differences of the audio effects depending on the gender of the speaker

Effects	Transformations								
	Happy			Sad			Afraid		
	low	medium	high	low	medium	high	low	medium	high
**Pitch**									
shift, *cents*	+ 29.5	+ 40.9	+ 50.0	−39.8	−56.2	−70.0	–	–	–
**Vibrato**									
rate, *Hz*	–	–	–	–	–	–	8.5	8.5	8.5
depth, *cents*	–	–	–	–	–	–	26.1^*M*^ 13.7^*F*^	33.8^*M*^ 20.2^*F*^	40.0^*M*^ 33.0^*F*^
**Inflection**									
duration, *ms*	500	500	500	–	–	–	500	500	500
min., *cents*	−144.8	−158.9	−200	–	–	–	− 109.3^*M*^	− 141.0^*M*^	− 169.2^*M*^
							− 50.2^*F*^	− 101.1^*F*^	− 158.6^*F*^
max., *cents*	+ 101.3	+ 111.3	+ 140	–	–	–	+ 109.3^*M*^	+ 141.0^*M*^	+ 169.2^*M*^
							+ 50.2^*F*^	+ 101.1^*F*^	+ 158.6^*F*^
**Filter**									
cut-off, *Hz*	> 8000	> 8000	> 8000	< 8000	< 8000	< 8000	–	–	–
slope, *dB/octave*	+ 5.8	+ 6.6	+ 9.5	−7.8	−9.6	−12	–	–	–

### Methods

#### Participants

The validation studies of the emotional voice effects were carried out in four languages: French, English, Swedish and Japanese, in IRCAM (France), University College London (UK), Lund University (Sweden) and Waseda University (Japan). Participants in the study comprised 20 native French volunteers (mean age = 25.4 years, SD = 4.9, ten females), 27 native British English volunteers (mean age = 26.1 years, SD = 5.6, 17 females), 20 native Swedish volunteers (mean age = 28.1 years, SD = 5.3, ten females), and 20 native Japanese volunteers (mean age = 21.1 years, SD = 1.4, 10 females). Two female English participants were excluded because they did not satisfy age (18–40 years) or language requirements (participants had to be a native speaker of the test language). Furthermore, responses of one female English volunteer were not recorded during the emotion recognition task (see below) due to technical problems. Volunteers were recruited through local databases and mailing lists in the respective countries and were financially reimbursed for their participation. The study was approved globally by the IRB of the French Institute of Health and Medical Research (INSERM), as well as locally by the departmental review boards of University College London, Lund University and Waseda University. All participants gave written informed consent.

### Procedure

To test the criteria of recognizability, naturalness, and control of intensity, participants performed three consecutive tasks: A naturalness rating task, an emotion recognition task, and an intensity rating task. Participants always performed all three tasks in the aforementioned order to avoid an interference effect of the recognition of the emotional transformations on naturalness ratings. We ran these validation experiments in the four different languages to address the fourth requirement of multicultural validity. Together, the three tasks took approximately 1 h to complete.

The voice stimuli were presented through closed headphones (Beyerdynamics, DT770, 250 Ohm), with the sound level adjusted by the participant before the start of the experiment. Once the first task started, the sound level stayed the same throughout the entire duration of the experiment. An Apple MacBook Pro running PsychoPy (Peirce 2007) was used to control stimulus presentation and the recording of responses.

#### Emotion recognition task

In each trial, participants listened to two utterances of the same sentence and the same speaker. The first utterance was always the neutral recording and the second utterance was either the same recording unprocessed (neutral condition), or processed with one of the emotional transformations. We only used the neutral recordings in this task, the human-produced emotional expressions were used in the other two tasks described below. Participants compared the two utterances in order to indicate in a forced choice task whether the second extract, compared to the first, sounded happy, sad, afraid, neutral. Additionally, a “none of the above” label was included and participants were asked to choose this option whenever they heard a difference that did not fit one of the other response labels (e.g., because the voice did not sound emotional at all, or because it sounded more like another emotion or a mixture of different emotions). Participants could listen to the voices as many times as necessary to make their judgment before proceeding to the next trial.

#### Naturalness task

In this task, participants heard one emotional utterance, either human-produced or modified, per trial and rated the naturalness of the voice on a continuous scale anchored by “very artificial/not at all natural” and “not at all artificial/very natural” (1–100). At the start of the trial, an empty scale without slider was presented. The slider appeared after the participant clicked for the first time on the scale and could be re-positioned until the participant clicked on the “validate” button. Prior to the experiment, participants were told that some of the utterances were human-produced and that others had been manipulated by a computer algorithm. As in the decoding task, participants could listen to each audio clip as many times as needed to make their judgment.

#### Intensity task

In this task, as in the naturalness task, participants heard either a modified or a human-produced voice. In each trial the intended emotion label was presented on the screen and participants judged the emotional intensity on a continuous rating scale (1–100) anchored by “not at all happy/sad/afraid” and “very happy/sad/afraid”. In addition, participants rated the loudness (subjective sound intensity) of the utterance to avoid confusion between the emotional intensity and other perceived acoustic characteristics that are not necessarily related to the intensity of the emotion. Loudness ratings were not further analyzed.

### Data analysis

We calculated the mean ratings of naturalness and intensity for the naturalness and intensity tasks. In addition, we computed mean accuracy scores for the emotion recognition task. To take possible response biases in the recognition task into account, we calculated the unbiased hit rates (*H*_*u*_) and individual chance proportions (*p*_*c*_) for each participant (Wagner 1993). Unbiased hit rates take a value between zero and one and take into account how often an emotion is identified correctly, as well as the total number of times that an emotion label is used. *H*_*u*_ therefore comprises a measure of both the sensitivity and the specificity of each participant’s performance. We then compared the arcsine transformed *H*_*u*_ and *p*_*c*_ by means of paired *t* tests (Holm-Bonferroni corrected).

As a measure of effect size and for easier comparison with other studies conducted on different numbers of stimulus and response categories, we also report the proportion index (*pi*). The *pi* expresses the raw (biased) hit rate transformed to a standard scale where a score of 0.5 equals chance performance and a score of 1.0 represents a decoding accuracy of 100% (Rosenthal and Rubin 1989).

Furthermore, unbiased hit rates and naturalness and intensity ratings were analyzed with an ANOVA and significant effects were followed by post hoc multiple comparisons (Tukey’s honestly significant difference, HSD, *α* = .05).

## Results

### Emotion recognition task

**Fig. 3:**
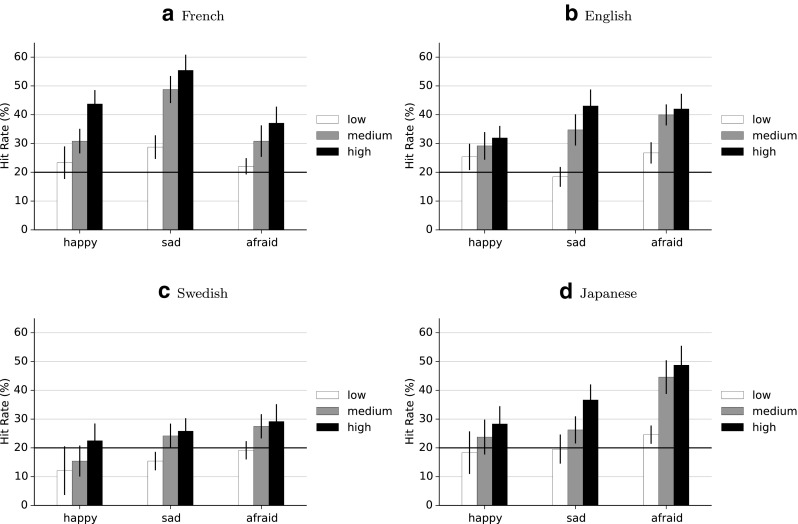
Raw hit rates. French **(a)**, English **(b)**, Swedish **(c)** and Japanese **(d)** raw accuracy scores for three emotions at the nominal level (*‘high’*) and two lower intensity levels, error bars represent SEM, black line represents chance level (20%)

**Fig. 4:**
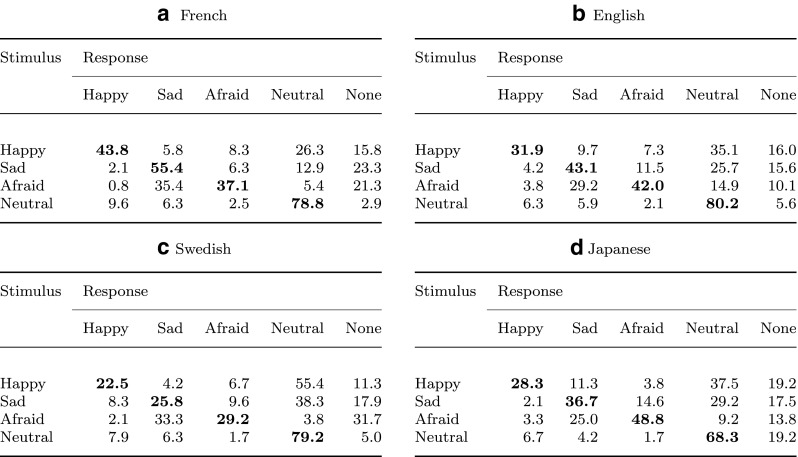
Confusion matrices. French **(a)**, English **(b)**, Swedish **(c)**, and Japanese **(d)** confusion matrices showing the distribution of responses (in %) at the nominal level. Diagonal cells in *bold* indicate correct responses

Raw hit rates for all intensity levels and all languages are shown in Fig. 3, where chance performance is 20%. The raw scores for the nominal level are represented in confusion matrices to provide some insight in the error patterns in the participants’ responses (Fig. 4). Paired *t* tests of the unbiased hit rates at the nominal level against the individual chance proportions showed that all three emotional effects were correctly recognized at rates above the individual chance level in all four languages (all *p*s < .01, Holm-Bonferroni corrected). See Table 3 for the statistical values.

**Table 3:** Emotion recognition scores, four languages

		Biased		Unbiased			
		*H*_*b*_	*pi*	*H*_*u*_	*p*_*c*_	*df*	*t*
FR	Happy	43.8	.76	.34	.042	19	6.2^∗∗∗^
	Sad	55.4	.83	.32	.061	19	5.5^∗∗^
	Afraid	37.1	.70	.28	.035	19	5.6^∗∗^
EN	Happy	31.9	.65	.31	.042	23	4.6^∗∗^
	Sad	43.1	.75	.23	.053	23	4.9^∗∗^
	Afraid	42.0	.74	.31	.039	23	6.1^∗∗∗^
SW	Happy	29.2	.62	.19	.047	19	3.7^∗^
	Sad	22.5	.54	.14	.051	19	2.9^∗^
	Afraid	25.8	.58	.21	.031	19	4.2^∗^
JP	Happy	28.3	.61	.26	.049	19	5.2^∗∗^
	Sad	36.7	.70	.21	.049	19	3.5^∗^
	Afraid	48.8	.79	.38	.043	19	5.8^∗∗^

FR = French; EN = English; SW = Swedish; JP = Japanese; *H*_*b*_ = raw hit rate (%); *pi* = proportion index; *H*_*u*_ = unbiased hit rate; *p*_*c*_ = chance proportion; *df* = degrees of freedom; *t* = *t*-score; *p* values are Holm- Bonferroni corrected. Please note that chance performance is 20% for *H*_*b*_ and .50 for *pi*. ^∗^ *p* < .01, ^∗∗^ *p* < .001, ^∗∗∗^ *p* < .0001.

A two-way ANOVA of the unbiased hit rates at the nominal level with emotion as within-subject variable and language as between-subject variable showed a main effect of language, *F*(3,80) = 2.87, *p* < .05. Tukey’s HSD post hoc test showed that this effect was driven by the Swedish participants who scored lower than both French and Japanese participants. There was also a main effect of emotion, *F*(2,160) = 3.68, *p* < .05, with sad (*H*_*b*_: M = 39.4%) and afraid (*H*_*b*_: M = 38.4%) scoring higher than happy (*H*_*b*_: M = 33.3%) - although Tukey’s HSD post hoc tests did not confirm a difference in performance between the three emotions. There was no significant interaction effect between language and emotion, *F*(6,160) = 1.80, *p* = .10.

### Naturalness rating task

**Fig. 5:**
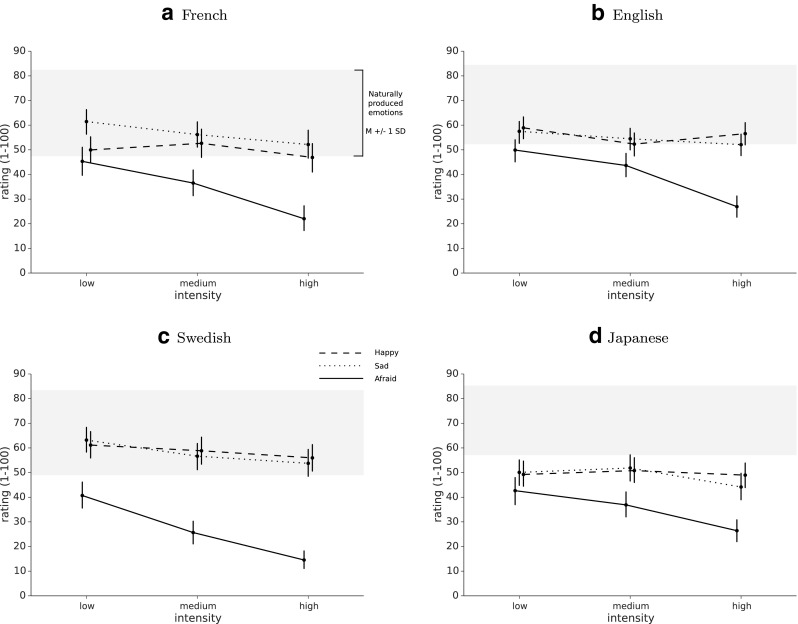
Naturalness. French **(a)**, English **(b)**, Swedish **(c)**, and Japanese **(d)** naturalness ratings for three emotions at three intensity levels compared to unmodified voices (*grey*: mean ± 1 SD), *error bars* represent 95% confidence intervals

We used the naturalness ratings of the human-produced emotional speech in our set of stimuli to position the emotional transformations against typical, authentic speech. Mean ratings for each emotional transformation at the three intensity levels are presented in Fig. 5a for all four languages, compared to ratings of human-produced voices.

Two-way mixed ANOVAs, one for each emotion, with intensity as within-subject variable and language as between-subject variable revealed no effects for the happy transformation (language: *F*(3,81) = 1.87, *p* = .14; intensity: *F*(2,162) = 1.48, *p* = .23; language × intensity interaction: *F*(6,162) = 1.41, *p* = .21). The results for the sad transformation revealed only a main effect of intensity, *F*(2,162) = 12.45, *p* < .001, Post hoc Tukey’s HSD test showed that naturalness ratings were significantly lower for the high intensity level compared to the low intensity level. The analysis of the afraid transformation showed both a main effect of language, *F*(3,81) = 3.25, *p* < .05 and a main effect of intensity, *F*(2,162) = 102.12, *p* < .001, but no interaction effect, *F*(6,162) = 1.47, *p* = .19. Tukey’s HSD post hoc test revealed that Swedish participants rated the stimuli as less natural than English participants. Furthermore, the transformations at medium intensity levels were rated more natural than those at the lowest intensity level and less natural than transformations at nominal (strongest) intensity level.

Additionally, we present effect sizes and the probability of inferiority for each emotional transformation compared to the three human-produced emotions grouped together in Table 4. The probability of inferiority (POI) is calculated by subtracting the common language effect size statistic (McGraw and Wong 1992) from 100% and it represents the probability that an utterance drawn at random from the set of transformed voices has a higher naturalness rating than an utterance drawn at random from the set of human-produced emotional voices.

**Table 4:** Cohen’s *d* and probability of inferiority (POI) of the naturalness ratings for each emotional transformation compared to natural emotional voices

		French		English		Swedish		Japanese	
		Cohen’s *d*	POI (*%*)	Cohen’s *d*	POI (*%*)	Cohen’s *d*	POI (*%*)	Cohen’s *d*	POI (*%*)
Happy	low	0.86	27.6	0.54	35.1	0.29	41.9	1.51	14.3
	med	0.81	28.3	0.92	25.8	0.45	37.5	1.40	16.1
	high	1.08	22.2	0.75	29.8	0.64	32.5	1.42	15.8
Sad	low	0.21	44.1	0.67	31.8	0.18	44.9	1.44	15.4
	med	0.57	34.3	0.77	29.3	0.58	34.1	1.30	17.9
	high	0.85	27.4	1.04	23.1	0.78	29.1	1.69	11.6
Afraid	low	1.20	19.8	1.18	20.2	1.58	13.2	1.68	11.7
	med	1.71	11.3	1.43	15.6	2.77	2.5	2.31	5.1
	high	2.66	3.0	2.43	4.3	3.82	0.3	2.87	2.1

At the nominal level, the mean natural ratings were 46.9 for happy, 52.2 for sad, and 22.0 for afraid, with 95% confidence intervals [39.5, 54.3], [46.5, 57.9], and [15.2, 28.8], respectively (in the French language, see Fig. 5a for complete results). The mean naturalness rating of the sad transformation fell within one standard deviation of the mean naturalness rating for the human-produced emotions (M = 64.9, SD = 17.5), meaning that POI = 27.4% of the human-produced stimuli were judged less natural than the effect (at nominal level). The mean rating for happy fell just outside of this range, with POI = 22.2% at nominal level. The afraid effect was rated as least natural (mean = 22.0, POI = 3%).

### Intensity rating task

We performed a separate two-way mixed ANOVA for each emotion, with intensity as within-subject variable and language as between-subject variable. For the happy transformation, there was a main effect of language, *F*(3,81) = 4.73, *p* < .01, but no main effect of intensity, *F*(2,162) = 0.58, *p* = .56, and no interaction effect, *F*(6,162) = 0.93, *p* = .48. Tukey’s HSD post hoc tests showed that Japanese intensity ratings were lower than in all three other population and that Swedish ratings were lower than French ratings.

For the sad transformation, there was no main effect of language, *F*(3,81) = 1.73, *p* = .17, but there was a main effect of intensity, *F*(2,162) = 8.30, *p* < .001. There was no interaction effect, *F*(6,162) = 1.52, *p* = .17. Tukey’s HSD post hoc tests failed to confirm any difference in intensity ratings between the three intensity levels.

**Fig. 6:**
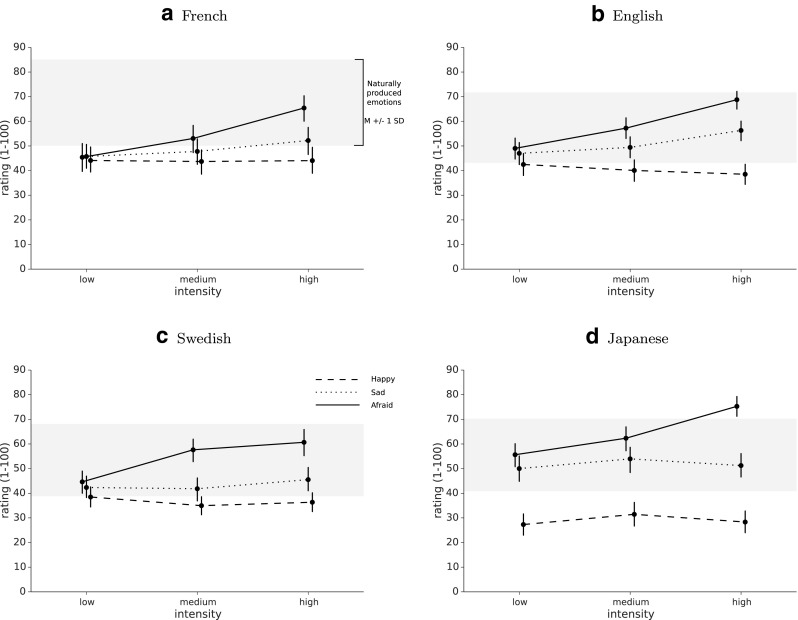
Intensity. French **(a)**, English **(b)**, Swedish **(c)**, and Japanese **(d)** intensity ratings for three emotions at three intensity levels compared to unmodified voices (*grey*: mean ± 1 SD), *error bars* represent 95% confidence intervals

Finally, for the afraid effect there was no main effect for language, *F*(3,81) = 1.76, *p* = .16, a main effect for intensity, *F*(2,162) = 86.34, *p* < .0001, and no interaction effect, *F*(6,162) = 1.27, *p* = .28. Tukey’s HSD post hoc tests showed that transformations at the weakest intensity level received lower intensity ratings than those at the medium intensity level and that both levels were rated as less intense than the strongest intensity level (Fig. 6).

## Discussion

We presented here a software tool that we have developed to make a neutral voice sound happier, more sad, or more afraid, by applying auditory transformations that operate in real-time at the infra-segmental level. In this study, we tested the following four requirements for the emotional transformations: (1) recognizability, (2) naturalness, (3) control of intensity, and (4) applicability in several languages.

### Recognizability

We tested the first requirement of emotion recognizability by means of a decoding task. The results show that French, English, Swedish and Japanese participants were able to decode these three emotional transformations with accuracies above chance level, with sad (39.4%) and afraid (38.4%) better recognized than happy (33.3%).

The fact that some transformations are more easily recognized than others could always be explained by algorithmic differences, in which one effect could be e.g., a “better-engineered” simulation of human-produced expression than another. However, because the happy and sad transformations largely rely on the same building blocks (pitch shift up or down, high- or low-shelf filter), we find this explanation unsatisfactory, and suggest that this difference is due to cognitive effects that would occur identically with human-produced expressions. It is well documented that, irrespective of language, some emotion displays are recognized more accurately than others, with negative emotions (such as anger or fear) often being more easily recognized than happiness (see e.g., Pell et al., 2009; Paulmann and Uskul 2014). It has been argued that recognizing potential danger (such as, here, the afraid transformation) is more adaptive than a non-threatening situation (see Öhman 2002, in the facial domain), whereas vocally expressed joy or happiness is especially strongly modulated by culture differences, even within a language group (Juslin and Laukka 2003).

While the accuracies obtained here obeyed the same type of pattern, and roughly fell within the range of decoding rates reported in other studies of human-produced speech (see e.g., the meta-study by Juslin and Laukka (2003). *pi*(happy) = 0.66 (this study, all languages averaged) vs. 0.51–1.0 (Juslin & Laukka); *pi*(sad) = 0.71 vs. 0.80–1.0; *pi*(afraid) = 0.70 vs. 0.65–1.0), they were still relatively low compared to typical performance (e.g., the mean hit rates reported in Scherer et al. (2011), H(happy) = 54%, H(sad) = 69%, H(afraid) = 62.4%). Moreover, neutral (unmodified) expressions were labeled correctly more often than any of the transformed emotions.

Several factors may explain these results. First, the difference between emotion recognition accuracies in this study and other studies using acted emotional expressions are likely a consequence of the tool’s operating on only infra-segmental speech features (and not e.g., on speech rate and pitch contour). The emotional tone of the transformed voices is therefore more subtle—and expressed with a more restricted set of cues—than acted emotional expressions. It is therefore in line with expectation that, by manipulating only a subset of the acoustic markers involved in the vocal expression of emotions, the decoding accuracy should be reduced and biased towards the neutral label.

Second, a forced-choice test, as we used in this study, may bias performance because of the limited response options (for further discussion on this topic see e.g., Banse and Scherer 1996; Scherer et al., 2003). However, we found that this test would be best suited to compare results across several languages (see below for further discussion of cross-cultural results). The response option *“None of the above”* was added to avoid forcing participants too much towards a certain emotion label. Additionally, we analyzed the results as unbiased hit rates to control for possible asymmetries between response categories.

Third, the data of all languages show a confusion between the afraid and sad labels, where an afraid voice is often identified as a sad one. Because the vibrato effect is a particularly salient component of the afraid transformation, we could speculate that this may have been perceived as a trembling voice of someone who is on the verge of crying, which would explain the frequent use of the sad label. This confusion between “cold” and “hot” sadness (low or high arousal) has in particular already been noted in the Japanese language (Takeda et al. 2013), and could explain parts of our results.

Fourth, the high performance for neutral utterances is likely due to both the subtlety of the emotional effects and the fact that each trial comprised a neutral reference voice. As a result the response strategy is slightly different for the neutral vocalizations, which would involve reporting the absence of any auditory transformation. Conversely, when a transformation is perceived, the next decision to be made is then more subtle because the appropriate label for the transformation should be chosen out of four options. We would argue that this could lead to a decrease in performance. Furthermore, the use of a neutral reference voice brings up another issue worthy of discussion, because studies using a similar paradigm (i.e., comparing a neutral voice to a pitch-shifted voice) found that pitch influences the perception of physical dominance and traits such as leadership capacity and trustworthiness for example (Klofstad et al., 2012, 2015; Vukovic et al., 2011). Because some of the emotional transformations in DAVID also use pitch-shifting, we cannot be certain that these acoustic manipulations are exclusively changing the emotional tone of the voice. So even though the instructions in this experiment involved a mapping of acoustic features onto emotions, we cannot rule out that participants perceived differences in personality traits or physical characteristics as well.

Finally, we cannot exclude the possibility that the semantic content has influenced the recognizability of the emotional transformations. In this study we included only semantically neutral sentences because we wanted to use the same sentence for each of the emotional transformation, trying to avoid a bias towards a certain emotion caused by the content of the phrase. However, it could be that a neutral sentence such as “the plane is almost full” does create a mismatch when pronounced with a voice that sounds afraid. Indeed, it has been shown that a mismatch between sentence content and the voice quality (e.g., negative sentence content and a voice quality expressing positive valence) can render utterances to be perceived as emotionally ambiguous (Aylett et al. 2013).

### Naturalness

To evaluate the transformations’ ability to pass as authentic speech, we asked participants to rate the naturalness of both transformed voices and human-produced emotional expressions. While the effects were generally rated as less natural than human-produced speech, naturalness ratings for happy and sad still fell within one standard deviation of the mean ratings for authentic speech, with one fourth to one third of our human-produced stimuli being rated as *less* natural than our effects. Moreover, naturalness ratings of these two emotions did not differ significantly across the four different languages and across the three intensity levels. Naturalness for the afraid effect was more problematic, and behaved like happy and sad only at the weakest intensity levels. In all four languages, stronger intensity levels significantly lowered the naturalness ratings of the afraid effect.

The interpretation of these results deserves caution. First, in our view, this task is not testing for people’s maximum capacity to recognize the manipulation, but for typical performance. In our view, there is always a situation where DAVID will fail. For instance, when one can compare an original recording with several of its transformations, it would not be hard to notice that transformations are bound to be the outcome of the system when the original prosody is exactly reproduced. What our data shows is that, at least in some situations, some natural sentences will be judged as equally or less natural than the transformations produced by DAVID. In our experience, the acceptance of DAVID-transformed speech as authentic cases of emotional speech is heavily dependent on context. For instance, in our recent study of vocal feedback where we instructed participants to read a text out loud while the effect was gradually increased without their knowing, only 14% of the participants reported detecting an artifact with their voice (Aucouturier et al. 2016). In contrast, had participants been instructed before the experiment about a potential voice manipulation, it is likely that this proportion would have been larger.

Second, it should be noted that the naturalness ratings of human-produced voices are not concentrated around the high end of the scale, showing that even authentic speech can be judged as not very natural. The relatively low ratings of human-produced voices in our study are likely due to the fact that participants were informed that some of the presented voices were computer manipulated. While it could be argued that such framing artificially reduced the baseline to which we compare the transformations, we believe it would be very difficult to elicit reliable responses without explicit instructions of what we mean by naturalness (i.e., here ”not artificially manipulated”). Indeed, judging a recording as ”natural” could be construed alternatively as evaluating the authenticity of the emotion (“is it sincere or simulated”), the match between an emotion and the sentence’s verbal content (“is it natural to be happy about an alarm clock”), or a rating of how well-acted the emotion was. Besides, it is not clear why such a paradigm should not also reduce the naturalness ratings of the manipulated recordings themselves.

Finally, the low naturalness scores of the afraid transformation at high intensity deserves a special mention. It is possible that this is a consequence of the vibrato effect used in this transformation, which may have provided a salient cue when compared to non-manipulated voices, either because it was too systematic or because it created occasional artifacts. It is to be noted that, in an alternative A/B testing paradigm reported in Aucouturier et al. (2016), the same effect was not discriminated from human-produced speech above chance-level. Rather than arguing whether one specific effect is “natural” or not, we hope that, by presenting effect sizes and probabilities of inferiority in each configuration, each reader can judge for themselves whether the tool is useful and appropriate for their own research.

### Control of intensity

To test whether the emotional intensity of the transformation could be controlled, we asked participants to evaluate the degree of emotional expression of each voice on a continuous scale, presenting both human-produced and transformed utterances at three different intensity levels. Our results show that, irrespective of language, both the angry and sad transformations were rated as more intense as we increased parameter levels. On the other hand, the intensity of the happy transformation did not seem to change for different parameter levels, for neither language. More generally, all transformations show a clear inverse relation between naturalness and intensity (the more intense, the less acceptable as authentic speech), and the choice of one particular configuration should follow which of these two factors is most important in each experimental context.

The lack of change for the happy effect is interesting as the different intensity levels do change recognition rates: it appears that, as we increase the depth of the pitch change and the amount of high frequencies in voice, transformed speech becomes more recognizably, but not more strongly, happy. This is especially surprising as this seems to hold in all four languages tested here, and the same effect does not seem to occur in the sad transformation, which yet uses symmetrical manipulations of pitch and spectral shape. Human actors have notorious difficulty manipulating the intensity of their emotional expression without a confounding variation of acoustic intensity or pitch (Juslin and Laukka 2001; Ethofer et al. 2006). Consequently, the psychoacoustics of emotional intensity (e.g., what makes a happy voice happier) is still unknown to a large degree. It would be interesting, with DAVID, to selectively manipulate the acoustical depth of various parameters (e.g., pitch shift independently from RMS energy), and examine how these parameter changes influence perceived emotional intensity.

One methodological limitation in this task is the fact that we normalized the sound level so that the stimuli were perceived with the same loudness for each intensity level and across the whole experiment. Previous studies have shown that loudness is an important cue of arousal in speech and nonverbal vocalizations (e.g., Lima et al., 2013; Juslin and Laukka 2001) and it is likely that changing this parameter would have an effect on the intensity ratings.

Taken together, these results warrant further investigation of the respective contribution of different acoustical characteristics to emotional intensity. One conservative conclusion is that the tool does not appear ideally suited to controlling the emotional intensity of happy vocalizations, in its current form.

### Intercultural applicability

Intercultural differences in the perception of vocal emotions, and emotional expression in general, are widely documented (for a review see e.g., Elfenbein and Ambady 2002; Scherer et al., 2011). The present set of tasks, conducted in four languages, depart a little from standard paradigms in that they are neither a test of cross-cultural universality, because the stimuli used in the four participant groups are not the same (e.g., Biehl et al., 1997), nor a test of intercultural differences (e.g., Elfenbein and Ambady 2002), because both speakers and decoders belong to the same cultural group. What these results address is the cross-cultural validity of the acoustic cues on which DAVID operates: participants in each cultural group listened to voices produced in their own language, albeit transformed with a unique algorithm applied identically to all languages.

Our results, like most previous studies, point at the co-existence of both universal cues of emotional expression and culturally learned display rules. On the one hand, the three emotional transformations were recognized above chance levels in all languages. On the other hand, language had an influence on performance in all of the three tasks. In the recognition task, Swedish participants scored lower than French and Japanese participants, irrespective of emotion. In the naturalness task, ratings for afraid were lower in the Swedish population than in the English. Finally, in the intensity task, happy was rated as less intense in Japan compared to all the other languages. Swedish intensity ratings of happy were also lower than French.

The fact that the same transformations were decoded above chance in four languages shows that the emotional cues manipulated in DAVID are not purely cultural. This may be a blessing of having to rely only on infra-segmental cues (for real-time constraints) and not manipulating supra-segmental aspects of speech such as intonation contours and rhythm, which Schröder (2001) has found can vary across language families and be difficult for outsiders to decode. Manipulating only pitch and spectral shape as we do here, if arguably accountable for relatively low recognition rates, at least appears to be a cross-culturally valid way to simulate emotional expression.

The amount of cross-cultural differences seen in our data in both recognition hit rates and intensity ratings is typical of other cross-cultural decoding studies with human-produced stimuli. Even on the same stimuli, different cultures perform differently and give different levels of intensity: ex. in Matsumoto and Ekman (1989), Americans gave higher absolute intensity ratings on facial expressions of happiness, anger, sadness and surprise than Japanese; in Biehl et al. (1997), non-western cultures gave higher intensity for fear, western cultures gave higher intensity for happy, and Japanese were worse in recognition of fear and sadness. Cross-cultural ratings of the perceived intensity of our transformations appear consistent with this pattern, with Japanese participants giving higher intensity for the afraid transformation, and English, French and Swedish participants giving higher intensity for the happy transformation.

Several factors may explain such differences in the agreement and intensity levels across cultures. First, the display rules of a given culture shape its members’ mental representations of emotions, such as the intensity level of emotional prototypes (Engelmann and Pogosyan 2013) or the accuracy of their decoding (Biehl et al. 1997). For instance, it is possible that lower intensity levels for fear and higher intensity for happiness are the cultural norm in Japan (which some have indeed argued, see e.g., Kitayama et al., 2000, 2006) and therefore that a given amount of expressivity (i.e., given parameter values) for these two emotions is judged, respectively, as higher and lower intensity by Japanese participants than by English, French or Swedish decoders.

Second, different cultures may have different cognitive strategies for judging the same emotion. For instance, when asked to judge the intensity of an emotion, Americans were found to rate the intensity of the external display of affect, while Japanese rated their perceived intensity of the subjective experience of the poser (Matsumoto 1999). Because the scale used in the intensity tasks confounded both constructs, it is possible that different cultures have in fact rated different aspects of the same stimuli, rather than differed in their rating of a common aspect.

Third, a difference in the level of agreement across cultures may also be explained by the translation of terms used as response categories (happy: joyeux, glad, yorokobi ; sad: triste, ledsen, kanashimi; afraid: inquiet, rädd, osore^3^). Even when terms are derived through back-translation, they may not be equivalent to the original, and in particular may not refer to the same facial or vocal expression. For example, shame and its common translation into Spanish (vergüenza), sadness and its common translation into Arabic (huzn), do not refer to emotions with identical features (de Mendoza et al. 2010; Kayyal and Russell 2012). In our data, Swedish participants were overall less accurate than French and Japanese participants, and notably mistook an afraid voice for a sad one more often than Japanese participants did. It is possible that these differences result from different boundaries between terms, and that the cues manipulated in the afraid effect spanned a larger proportion of the vocal expressions referred to as *ledsen* in Swedish than that referred to as “sad” or *triste* in French.

Finally, it remains possible that, while the cues manipulated in the transformations are cross-culturally valid, their algorithmic manipulation differed in salience depending on the phonetic characteristics of the language on which it is applied. Effects like vibrato for instance rely on the availability of relatively long voiced portions in phonemes (e.g., 250 ms for two cycles of an 8-Hz-vibrato to be perceived), and may not operate well on languages with a relatively large consonant/vowel ratio such as Swedish (Haspelmath 2005). Similarly, inflection added with the happy or afraid transformations may be more salient in languages that display comparatively flatter prosodic variations such as French. More research will be needed to understand how acoustic parameters that are optimal for emotion recognition depend on the phonetic characteristics of a given language, or even a given speaker. Until then, we encourage users to experiment with parameters beyond the values we suggested here, and to adapt them to the specificities of their experimental context.

### A note on applicability to other stimuli

DAVID has been developed to transform a neutral voice into an emotional voice, and not to transform an already emotional vocal expression into another type of emotion. While it would be interesting and of great use to transform a sad voice into a happy voice, this possibility was not addressed during the development of this tool and the execution of this study. Notably, because DAVID does not operate on supra-segmental cues, it is possible that sad voices made happier with DAVID present a conflicting mix of cues, with high pitch and increased high frequencies in the short-term but slow speech rate and decreasing prosody in the long term, and may not impart the intended emotion successfully.

Additionally, our objective has been to transform continuous, running speech and we ran this validation study in the context we believed the tool to be most useful in. As a result, one should be conscious that certain parameter settings that are acceptable in continuous speech may have a more pronounced effect in unconventional speech production, for example during the production of sustained vowels. Moreover, 96 (six speakers × four languages × four sentences) sentence-speaker combinations have been used in this study, resulting in a total of 288 different audio files that were transformed by DAVID (96 × 3 different recordings of each sentence). As such, any attempt at generalizing the results described here should be made with caution. All sound samples used in this study were made available for download from https://archive.org/details/DAVIDAudioFiles, and the tool is freely available at http://cream.ircam.fr. Again, even within a single language, we encourage users to experiment with parameters beyond the values we suggested here, and to adapt them to the type of stimuli used in their work.

## Applications

We presented here a new software tool that is able to transform a neutral voice into either a happy, sad, or afraid voice. The main innovation of our approach, differentiating it from previous work, is that the manipulations are *both* real-time and natural. As already mentioned, previous attempts at real-time voice modification typically aimed to create caricatural or gender changes. Conversely, naturalness has been typically achieved so far in an offline context (e.g., pre-recorded samples), and has rarely been formulated in the context of voice change (a natural first person), but rather that of synthesis (a natural third person). Here, we achieve both simultaneously. This opens up new possibilities for experimental paradigms, from controlling the emotional vocal expression of one participant to studying the effect of emotional prosody in group interactions. We list a selection of these applications below.

### Studies of emotional prosody

Traditional studies of emotional prosody typically rely on actor-produced impersonations of various emotions, a method plagued with problems of stereotypicality and parameter co-variation (Jürgens et al. 2015). Using DAVID, one can parametrically sample a large, generic space of emotional prosodic variations (e.g., all vibrato frequency between 1 Hz and 10 Hz) or selectively manipulate the depth of various parameters (e.g., pitch shift independently from RMS energy). This can be used e.g., to generate more ecologically valid templates of emotional expression for reverse correlation techniques (as done for faces -Mangini and Biederman, 2004), to produce a continuous range of stimulus difficulties, e.g., for iterative psychophysical procedures, or to produce cross-cultural stimuli that utilize exactly the same acoustic cues, with the same acoustical strength, rather than stimuli produced by native speakers of each language.

### Music cognition

Emotional expression in opera or pop music singers is an important element of music appreciation (Scherer 1995), and it has been also proposed that instrumental music can induce emotions by imitating expressive speech (Juslin and Västfjäll 2008). By using DAVID on multi-track music recordings, one can test these effects by generating vocal singing tracks with speech-like emotional transformations while keeping the musical background constant or applying the same acoustical transformations to musical instrument samples. In addition, vocal processing, such as automatic generation of a harmony voice to second the participant’s singing, is an important focus of the karaoke industry to improve customer enjoyment (Kato 2000). Using DAVID on singing voice, it is possible to test whether adding realistic emotional expressions in real time, possibly suited to the target song (e.g., a trembling transformation to a sad song) can enhance the singer’s (and his/her audience’s) appreciation of a performance.

### Social psychology

Social psychology often involves testing the effect on subsequent behavior of emotions displayed by one or several participants engaged in an social interaction, and do so either by instructing them explicitly to behave in certain way (e.g., Tice 1992) or leading them to display the emotion using a sophisticated cover story (e.g., Van Doorn et al., 2012). With DAVID, one can study causal relationships in such interactions by letting two participants talk (e.g., on the phone) and shifting their voices in congruent or incongruent directions, without such demand effects. This procedure can be used e.g., to study emotional stereotypes (Neuberg 1989), judgments of willingness to cooperate (Van Doorn et al. 2012), or the impact of emotional processes on group productivity (Parker and Hackett 2012).

### Emotion regulation

Emotion regulation paradigms often involve using expressive language or facial gestures while recollecting personal events or writing about oneself (e.g., Slatcher and Pennebaker 2006; Siegman and Boyle 1993). In such paradigms, it is often difficult to disentangle the role of emotional re-engagement from the effects of factual recollection and language production. Using DAVID, experimenters could ask participants to recollect or read out their expressive writing while manipulating the emotional tone of their voice in a congruent or incongruent direction, testing e.g., if the impact of emotional memories is attenuated when heard with a non-congruent tone of voice. This approach of mood induction via one’s own voice may be of particular interest in situations where self-referential processing is altered, such as with depressed patients (Grimm et al. 2009) or traumatic memories (Richards et al. 2000).

### Decision-making

Building on Damasio’s somatic marker hypothesis (Damasio 1994), research has found that giving participants false heart-rate feedback (Shahidi and Baluch 1991), instructing them to display a smile (Arminjon et al. 2015) or letting them experience physical pain Xiao et al. (2015) would change their judgments related e.g., to moral vignettes or their confidence in their own behavior. With DAVID, one can let participants read a text or recall a memorized event out loud while their voice is made to sound more or less positive, and test whether voice functions as a somatic marker for their decisions and judgments, without any demand effect. If one stimulus is read with a sad voice and the other with a happy voice, the prediction would be that participants be more negatively oriented to the one they have read with a negative sounding voice.

### Emotional monitoring

In the emotional vocal feedback study by Aucouturier et al. (2016), participants were not informed about the manipulation prior to the experiment. Their results showed that participants did not detect the manipulation, and did not compensate for the acoustical changes in their own vocal production. Using DAVID, it is possible to further explore these discrepancies, for instance testing under which circumstances (different types of social interactions versus self-focused out-loud reading) people may or may not adapt their speech in response to perturbed vocal feedback (see e.g., Behroozmand et al., 2012), or exploring the contextual requirements for accepting the manipulations (see e.g., Lind et al., 2014). For instance, it might be that a happy voice transformation is more easily accepted in a friendly social interaction than in an extremely hostile one.

### Augmentative and alternative communication devices

Text-to-speech technologies on augmentative and alternative communication (AAC) devices do not typically allow much customization of e.g., identity or emotional tone of voice (Mills et al. 2014; Pullin and Hennig 2015), which limits the communication repertoire, and in turn, technology adoption and social integration. Using DAVID, it would be possible to transform the output of text-to-speech synthesis in real-time to match the subjective emotional or physiological state of users of such devices, in a similar fashion as recent experiments with the musical sonification of physiological variables for individuals who are otherwise unable to communicate (Cheung et al. 2016).

## Footnotes

1 “Da Amazing Voice Inflection Device”, DAVID was so named after Talking Heads’ frontman David Byrne, whom we were privileged to count as one of our early users in March 2015.

2 An increase of 50 cents is equivalent to a ∼2.93% change of F0.

3 In more details, response categories in the recognition task for the French group in fact used the English terms (happy, sad, afraid) instead of the French equivalents, but were defined in the instructions using the equivalent French terms cited here; Response categories used in both the Swedish and Japanese groups were the Swedish and Japanese terms cited here
